# Delta-like 4 mRNA is regulated by adjacent natural antisense transcripts

**DOI:** 10.1186/s13221-015-0028-9

**Published:** 2015-03-24

**Authors:** Keguo Li, Tamjid Chowdhury, Padmanabhan Vakeel, Christopher Koceja, Venkatesh Sampath, Ramani Ramchandran

**Affiliations:** Department of Pediatrics, Medical College of Wisconsin, 8701 Watertown Plank Road, Milwaukee, WI 53226 USA; OBGYN, Medical College of Wisconsin, 8701 Watertown Plank Road, Milwaukee, WI 53226 USA

**Keywords:** Non-coding RNA, Delta-like4, Vascular, Hemangiomas

## Abstract

**Background:**

Recent evidence suggests that a majority of RNAs in the genome do not code for proteins. They are located in the sense (S) or antisense (AS) orientation and, to date, the functional significance of these non-coding RNAs (ncRNAs) is poorly understood. Here, we examined the relationship between S and AS transcripts in the regulation of a key angiogenesis gene, Delta-like 4 (*Dll4*).

**Methods:**

Rapid Amplification of cDNA Ends (RACE) method was used to identify natural antisense transcripts in the *Dll4* gene locus in murine and human endothelial cells, referred to as Dll4 Anti-Sense (*Dll4-AS*). Messenger RNA (mRNA) levels of *Dll4* and *Dll4-AS* were quantified by real-time PCR. The function of *Dll4-AS* was investigated by overexpression and knocking down of *Dll4-AS*.

**Results:**

*Dll4-AS* comprises of three isoforms that map proximal to the *Dll4* promoter region. Expression patterns of *Dll4-AS* isoforms vary among different endothelial cell lines, but are always congruent with those of *Dll4*. A dual promoter element in the *Dll4* locus has been identified that controls the expression of both transcripts. Both *Dll4-AS* and *Dll4* are sensitive to cellular density in that higher cellular density favors their expression. Exogenous *Dll4* stimuli such as VEGF, FGF and Notch signaling inhibitor altered both *DLL4-AS* and *DLL4* expression suggesting co-regulation of the transcripts. Also, knocking down of *Dll4-AS* results in down-regulation of *Dll4* expression. As a consequence, endothelial cell proliferation and migration increases in vitro, and sprout formation increases. The regulation of *Dll4* by *Dll4-AS* was also conserved in vivo.

**Conclusion:**

A novel form of non-coding RNA-mediated regulation at the *Dll4* locus contributes to vascular developmental processes such as cell proliferation, migration and sprouting.

**Electronic supplementary material:**

The online version of this article (doi:10.1186/s13221-015-0028-9) contains supplementary material, which is available to authorized users.

## Background

Recent findings demonstrate that a new class of RNA arising from intergenic or introns of vascular specific genes participate in the regulation of angiogenesis, the growth of new blood vessels from existing vasculature [1-3]. These RNAs are referred to as non-coding RNAs (ncRNAs) because majority of ncRNAs in the genome do not code for proteins [4]. NcRNAs are classified as long (>200 bp) (lncRNAs) or short (<200 bp) (sncRNAs) based on their sizes [5]. Recent evidence suggests that they are located in the sense (S) or antisense (AS) orientation [6,7] and, to date, the functional significance of these ncRNAs is poorly understood. Previous work from our laboratory identified a non-coding RNA in the antisense direction to the *tie1* locus, which participates in the regulation of the *tie1* mRNA [1] during embryonic vascular development. However, we noted that only a small but significant proportion of embryos displayed *tie1* loss-of-function phenotype. Therefore, we hypothesized that compared to recessive genes, haploinsufficient genes such as *Vegf* [8] and *Delta-like4* (*Dll4*) [9] are tightly regulated during vascular development by antisense RNA. We focused here on *Dll4*, an arterial endothelial specific ligand for Notch1 receptor [10]. We investigated whether antisense RNA exists in the *Dll4* locus, and whether they had a functional relevance to *Dll4* mRNA regulation. Delta-like 4 (*Dll4*) is an arterial endothelial specific ligand for Notch1 receptor [10], and is a vascular-specific haploinsufficient gene [9], in that loss of one copy causes phenotype. *Dll4* plays a paramount role in angiogenesis; and altered *Dll4* levels during mouse development caused vascular malformations, leading to lethality [9]. In this study, we have identified lncRNAs at the *Dll4* locus. They are transcribed anti-sense to *Dll4*, and therefore, we refer to these transcripts as *Delta-like4* antisense (*Dll4-AS*). Both *Dll4* and *Dll4-AS* transcripts share a common promoter element in the *Dll4* genomic locus. Further, we identify that *Dll4-AS* regulates *Dll4* mRNA levels in vitro, and this regulation has functional consequences.

## Methods

### Identification of *Dll4-AS*

PolyA RNAs were isolated from mouse endothelial cell line, MS1 using a Poly(A)Purist Kit (Life Technologies, AM1916). FirstChoice RLM-RACE Kit (Life Technologies, AM1700) was used to obtain the full length sequences of *Dll4-AS*. The RACE primers were derived from the cDNA sequence of Gm14207 (Accession No. NR_030683). RACE primers were cctcttcccttaggagtgtgtcctctgt (5′ outer), aggtggcctctggttgtcttcatgt (5′ inner), ctcggcttttcctcatacctc (3′ outer) and tgtccactgtctggttgctc (3′ inner).

### Subcellular localization of *Dll4-AS*

NE-PER Nuclear and Cytoplasmic Extraction Kit (Thermo Scientific #78833) was used to separate nuclei and cytoplasm of MS1 cells. The separated two parts were subjected to TRIzol extraction, followed by reverse transcription. *Xist* served as positive control for nuclear compartment and *tRNA-Met* for cytoplasmic compartment. The primers were Xist-for, tgcgggttcttggtcgatgt, Xist-rev, cgcttgagatcagtgctggc; tRNA-Met-for, ggcccataccccgaaaac, tRNA-Met-rev, acgggaaggatttaaaccaa.

### Quantitative PCR

Total RNA from cultivated cells was extracted by TRIzol reagent followed by DNase I treatment for 2 h at 37°C. The DNA-free RNA was further purified using RNAeasy Mini kit (Qiagen, 74104). RNA concentrations were measured by Nanodrop (Thermo Scientific), followed by reverse transcription by SuperScript III (Life Sciences). Quantitative PCR was carried out with SYBR Green I in iQ5 Multicolor Real-Time PCR Detection System (Bio-Rad, 170–9780). The expression levels were normalized to internal βactin or *CD31*. Primers for mouse βactin, *Dll4-AS1*, *−AS2*, *−AS3*, total *Dll4-AS* and *Dll4* were: βactin-for, ctcttttccagccttccttct, βactin-rev, aggtctttacggatgtcaacg; AS1-for, ttctcaaaaactccgctgct, AS1-rev,ctctgctctttcccctcctc; AS2-for, atccgacgccttaacctttc, AS2-rev,ctccgttctgctcctattgc; AS3-for, cccgaaaccttgacttttca, AS3-rev, ccaccagaggataggagggta; ASt-for, gaggcaataggagcagaacg, ASt-rev, gccaggttgttcagtcaaga; Dll4-for, cagagacttcgccaggaaac, Dll4-rev, actgcagatgacccggtaag. Primers for human *CD31*, *DLL4* and *DLL4-AS* were: CD31-for, tgaacctgtcctgctccatc, CD31-rev, ccgactttgaggctatcttgg; DLL4-for, actgtgcccgtaacccttg, DLL4-rev, tggagaggtcggtgtagcag; and DLL4-AS-for, agatgccttgtgtgggacta, DLL4-AS-rev, cctctctcaactccaaatcctg.

### Promoter reporter gene assay system

DNA fragments mapped to *Dll4* promoter regions were cloned into pGL4.14 vector (Promega, E6691) using In Fusion cloning system (Clontech, 638909). pGL4.14 constructs containing different inserts were mixed with pRL-TK Vectors (Promega, E2241) at 20/1 for co-transfecting MS1 cells. Before luciferase activity was determined, the cells were re-plated onto 24-well plate so that the cellular density reached 90% confluence at the time point of assay. Following the addition of 200 μL 1X reporter lysis buffer into each well, the plate was placed at −80°C for 30 min and then equilibrated at room temperature. The cellular lysate was centrifuged at maximum speed for 1 min. Cleared lysates were used to measure luciferase and renilla activity on GloMax® 20/20 Luminometer (Promega, E5331) by Dual-Luciferase Reporter Assay System (Promega, E1910).

### Overexpression of *Dll4-AS*

pTracer™-CMV2 Vector (Life technologies, V885-01) was digested with EcoRV and NotI for sub-cloning *Dll4-AS* isoforms. *Dll4-AS1, −AS2* and *-AS3* were amplified from MS1 cDNA using Phusion DNA polymerase and the primers harbored NotI site at the 3′ end. The plasmid contained a separate cassette encoding green fluorescent protein (GFP) -Zeocin expression that allowed for stable selection of transfected cells. Transfection of the plasmids was performed according to the protocol of Lipofecatmine 2000 (Life technologies, 11668027). The cells were selected by Zeocin, and cells overexpressing *Dll4-AS* were tracked through GFP expression.

### *Dll4-AS* knockdown

Vector-Based miRNA and synthetic siRNA were used to knockdown *Dll4-AS* expression in both MS1 and EOMA cells. pcDNA6.2-GW/EmGFP-miR (Life technologies, K4936-00) was used to express miRNA targeting the common last exon of all *Dll4-AS* isoforms. The inside single-stranded DNA oligonucleotides encoding the target pre-miRNA and the complementary oligonucleotides for miR1 are tgctgaggttgttcagtcaagaacctgttttggccactgactgacaggttcttctgaacaacct and cctgaggttgttcagaagaacctgtcagtcagtggccaaaacaggttcttgactgaacaacctc, respectively. Those for miR2 are tgctgctctgattagatccattcaaggttttggccactgactgaccttgaatgtctaatcagag and cctgctctgattagacattcaaggtcagtcagtggccaaaaccttgaatggatctaatcagagc, respectively. The forward and reverse synthetic siRNAs for control, siRNA1 and siRNA2 are ggugagccguguagaguaatt, uuacucuacacggcucacctt, gguaggaggccugugauaatt, uuaucacaggccuccuacctt, ggaggccugugauaagguutt, aaccuuaucacaggccucctt, respectively.

### Immunostaining

MS1 cells were plated on sterile glass coverslips placed in the wells of a culture plate and allowed to adhere overnight. Cells on coverslips were transfected with siRNAs. 48 h later, the coverslips were removed from the well, washed in TBS [50 mM Tris · HCl (pH 7.4), 150 mM NaCl], and fixed in 4% (wt/vol) paraformaldehyde for 10 min before permeabilization in 0.2% (vol/vol) Triton X-100 for 5 min. Cells were blocked in PBS with 1% goat serum and 0.1% Tween for 1 h at room temperature before staining with goat anti-mouse DLL4 (R&D, AF1389). Following 45 min incubation, cells were washed in TBS, followed by incubation with a fluorescent-conjugated IgG secondary antibody for 30 min in dark. After staining, coverslips were mounted in VectaShield containing DAPI.

### Mouse studies

Care of the mice during experimental procedures was conducted in accordance with the policies of the Biomedical Resource Center, Medical College of Wisconsin, and the National Institutes of Health guidelines for the care and use of laboratory animals. Protocols had received prior approval from the Medical College of Wisconsin Institutional Animal Care and Use Committee. C57BL/6 mice were obtained from Charles River Laboratories (Franklin, CT). Six day-old C57BL/6 mouse pups were injected intraperitoneally with 1 mg/kg ultrapure LPS (Invivogen, CA) or saline and lungs were harvested after 18 h following sacrifice of animals. RNA was obtained from whole lung using the PureLink RNA kit from Life Technologies (Carlsbad, CA).

### Cell proliferation assay

Cell proliferation was performed using an ELISA kit (Roche # 11647229001). 5 × 10^3^ cells were inoculated into 96-well culture plate and cells were allowed to attach for 12 h. siRNA-lipid complexes containing 1 pmol mixed siRNA and 0.3 μl Lipofectamine RNAiMAX was added into each well. Medium was refreshed 48 h later and 10 μl BrdU labeling solution was added into it. 12 h later, the labeling medium was replaced by 200 μl FixDenat and incubated for 30 min at room temperature. The FixDenat was replaced by 100 μl anti-BrdU-POD working solution. 90 min later, the wells were washed 3 times with PBS. 100 μl substrate solution was added into each well. Photometric detection was performed 20 min later by SpectraMax 340PC384 Microplate Reader (Molecular Devices).

### Cell migration assay

EOMA cells were plated onto a 6-well plate. After the cells adhered to the plate surface, control or mixed *Dll4-AS* siRNA were introduced into the cells by Lipofectamine RNAiMAX. 48 h later, the cells were plated onto transwell inserts at 4 × 10^4^ cells/well in 500 μL of medium. The transwell inserts were then inserted into a 24-well plate containing 750 μL of medium. Cells were allowed to migrate at 37°C, 5% CO_2_ for 2 h. Cells were then fixed at 4% PFA at RT for 20 min, and were further stained for 5 min with crystal violet (Sigma) in 2% ethanol and then rinsed in water. The cells on the upper side of the inserts were removed with a cotton swab, and the cells on the lower side of the inserts that were counted under light microscopy. Data are expressed as the mean ± S.D. of 3 independent assays.

### Spheroid sprouting assay

MS1 cells in Dulbecco’s modified Eagle medium (DMEM) were suspended in hanging drops (300 cells/30 μL) on the underside of petridish lids. The hanging drops were incubated for 24 h to form spheroids. Harvested spheroids were suspended in 1.5% collagen gel, and the spheroids-containing collagen gel was rapidly transferred into 96-well plates pre-coated with the same collagen gel and allowed to polymerize at 37°C for 30 min. DMEM containing 30 ng/mL recombinant mouse VEGF was added to the plates to cultivate the cells for 7 days. Sprouting vessels were quantified under microscope by counting the sprouts that had grown out of each spheroid.

## Results

### Identification and characterization of *Dll4-AS*

**Figure 1:**
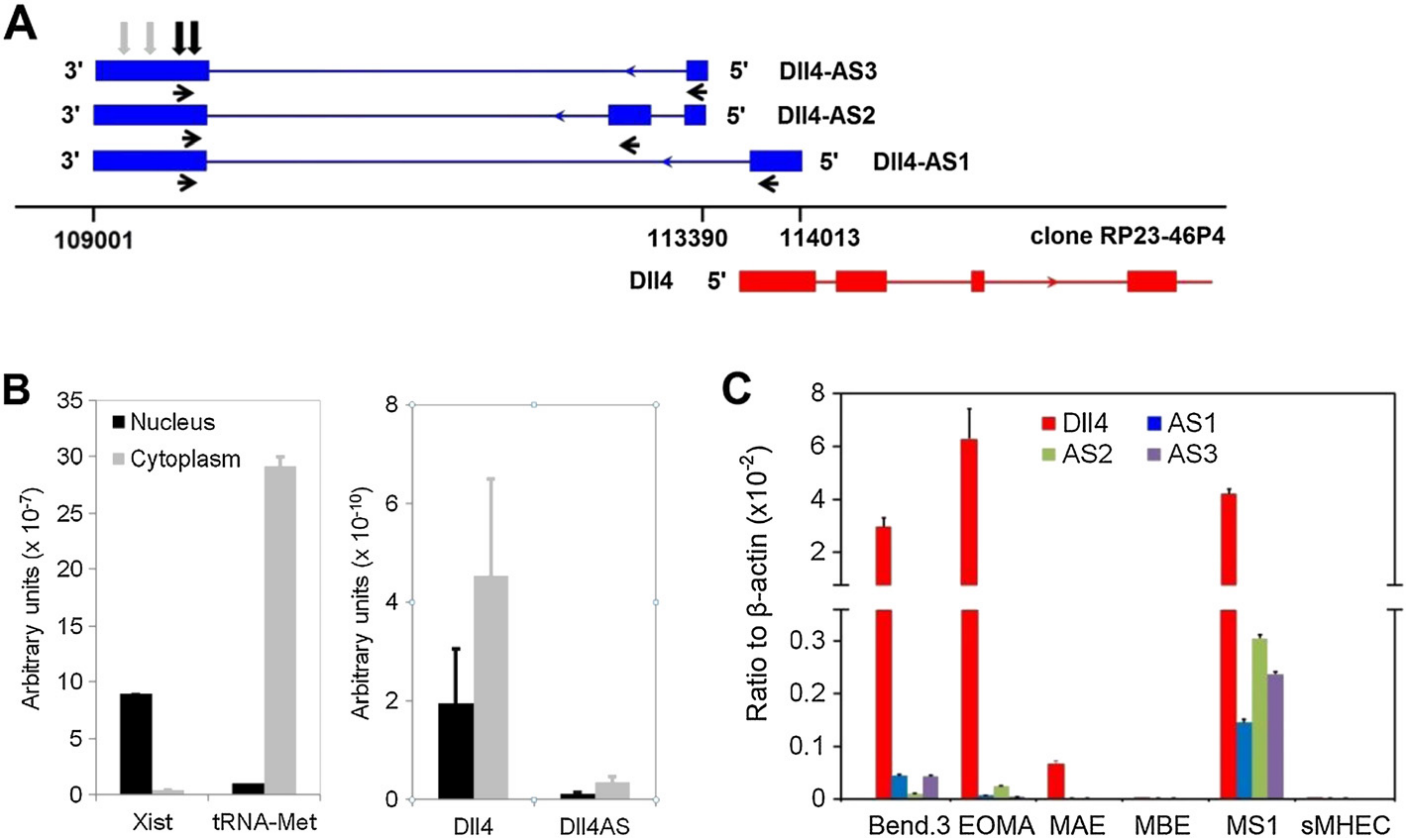
**Identification of** ***Dll4-AS*** **isoforms. (A)** RACE reactions mapped the *Dll4-AS* into *Dll4* promoter region. Blue and red arrowheads indicate transcriptional orientations of Dll4-AS and Dll4, respectively. Horizontal black arrowheads indicate following primers’ locations. Vertical black and grey arrows indicate following siRNA and miRNA targets, respectively. **(B)** *Dll4-AS* is enriched in cellular cytoplasm. **(C)** *Dll4-AS* expression coincides with *Dll4* in different cell lines. Bend.3: mouse endothelial cell from cerebral cortex; EOMA: mouse endothelial cell from hemangioendothelioma; MAE: Mouse Aortic Endothelial cell; MBE: Mouse Brain capillary Endothelial cell; MS1: mouse endothelial cell from pancreas; sMHEC: Mouse Heart Endothelial Cells.

We searched the mouse genome databases for non-coding RNAs at *Dll4* locus, and found a predicted gene 14207 (Gm14207, Accession No. NR_030683). A cDNA clone containing Gm14207 was identified from the mouse thymus, and because *Dll4* is expressed in both thymus and vasculature [11], we investigated the expression pattern for Gm14207 in endothelial cells (ECs). RT-PCRs based on the Gm14207 sequence was performed, and PCR products of different sizes were amplified in mouse pancreatic endothelial cell line MS1. Rapid Amplification of cDNA Ends (RACE) identified three transcripts antisense to the *Dll4* locus in mouse ECs (Figure 1). We named these transcripts Delta-like 4 antisense1 (*Dll4-AS1*), Delta-like 4 antisense2 (*Dll4-AS2*), Delta-like 4 antisense3 (*Dll4-AS3*) with lengths of 558 bp, 720 bp and 687 bp, respectively. The sequences are deposited in GenBank (Accession numbers: KP171170, KP171171 & KP171172). To localize *Dll4-AS* transcripts in cellular compartments, we used NE-PER Kit (Thermo Scientific) to separate cytoplasmic and nuclear RNAs, and performed RT-PCR using primers located in the common region (Figure 1A). *Dll4-AS* transcripts were localized in both cytoplasm and nucleus of mouse ECs (Figure 1B). Subsequently, we examined the expression levels of *Dll4-AS* and *Dll4* in different mouse endothelial cell lines (Bend.3: mouse endothelial cell from cerebral cortex; EOMA: mouse endothelial cell from hemangioendothelioma; MAE: Mouse Aortic Endothelial cell; MBE: Mouse Brain capillary Endothelial cell; MS1: mouse endothelial cell from pancreas; sMHEC: Mouse Heart ECs). We found that *Dll4-AS* level differs dramatically among the tested cell lines. However, comparing across cell lines the trend is the same in that *Dll4-AS* levels are congruent with *Dll4* (Figure 1C). To identify human *DLL4-AS*, we searched for transcripts at human *DLL4* promoter region in the UCSC Genome Browser. Item 3619762 of Affymetrix Exon Array from ENCODE/UW was retrieved and validated by RT-PCR. The transcript was extended to 768 bp by tiling PCR (Additional file 1: Figure S1).

### *Dll4-AS* shares a common promoter with *Dll4*

**Figure 2:**
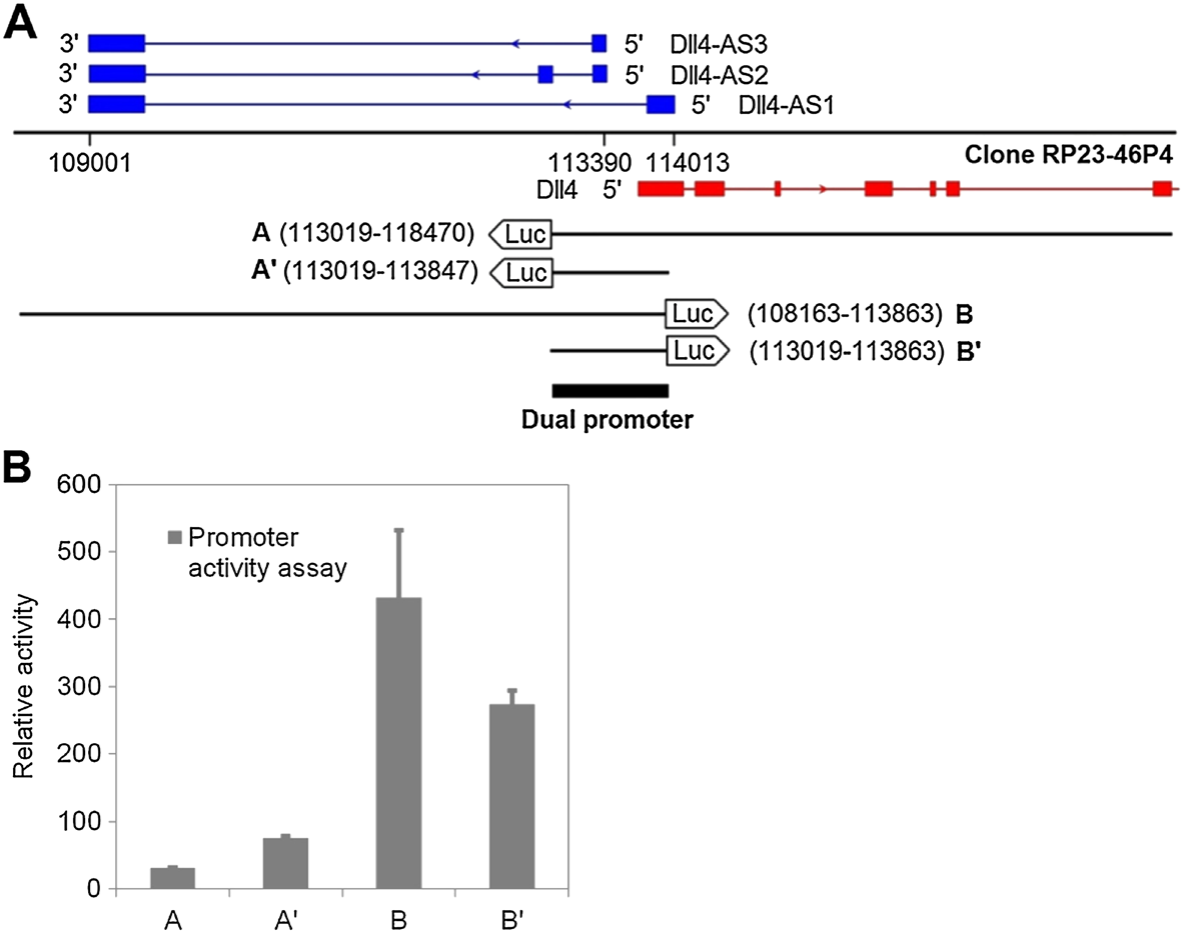
**Identification of** ***Dll4-AS*** **promoter. (A)** Genomic fragments aligning with both *Dll4* and *Dll4-AS* transcription start sites were cloned into a promoter reporter vector, pGL4.14. **(B)** These constructed vectors were mixed with 1/20 (molar ratio) Renilla vector, which serves as internal control for cell transfection. The transfected MS1 cells were lysed 48 h later for luminescence reading.

To identify the genomic element that drives *Dll4-AS* expression, we cloned DNA fragments near the transcription start site of *Dll4-AS* into a promoter reporter vector, pGL4.14 (Figure 2A). Those fragments were placed in different directions, corresponding to either *Dll4-AS* or *Dll4* transcription into the vector, and co-transfected with renilla construct into MS1 cells. Fragments A and A’ were in the antisense (*Dll4-AS*) direction, while fragments B and B’ were in the *Dll4* sense direction. Lysates were generated, and *luciferase* gene readouts were measured. Fragment B and B’ showed the most activity when compared to A and A’. Interestingly, A’ and B’ is the same fragment in opposite direction, and show promoter activity in both directions. These results indicate that the promoter of *Dll4-AS* is the same region as that of *Dll4* (Figure 2B), and imply that the two transcripts share the same promoter region, and drives RNA transcription in both directions.

### *Dll4-AS* expression is concomitant with *Dll4* mRNA expression

**Figure 3:**
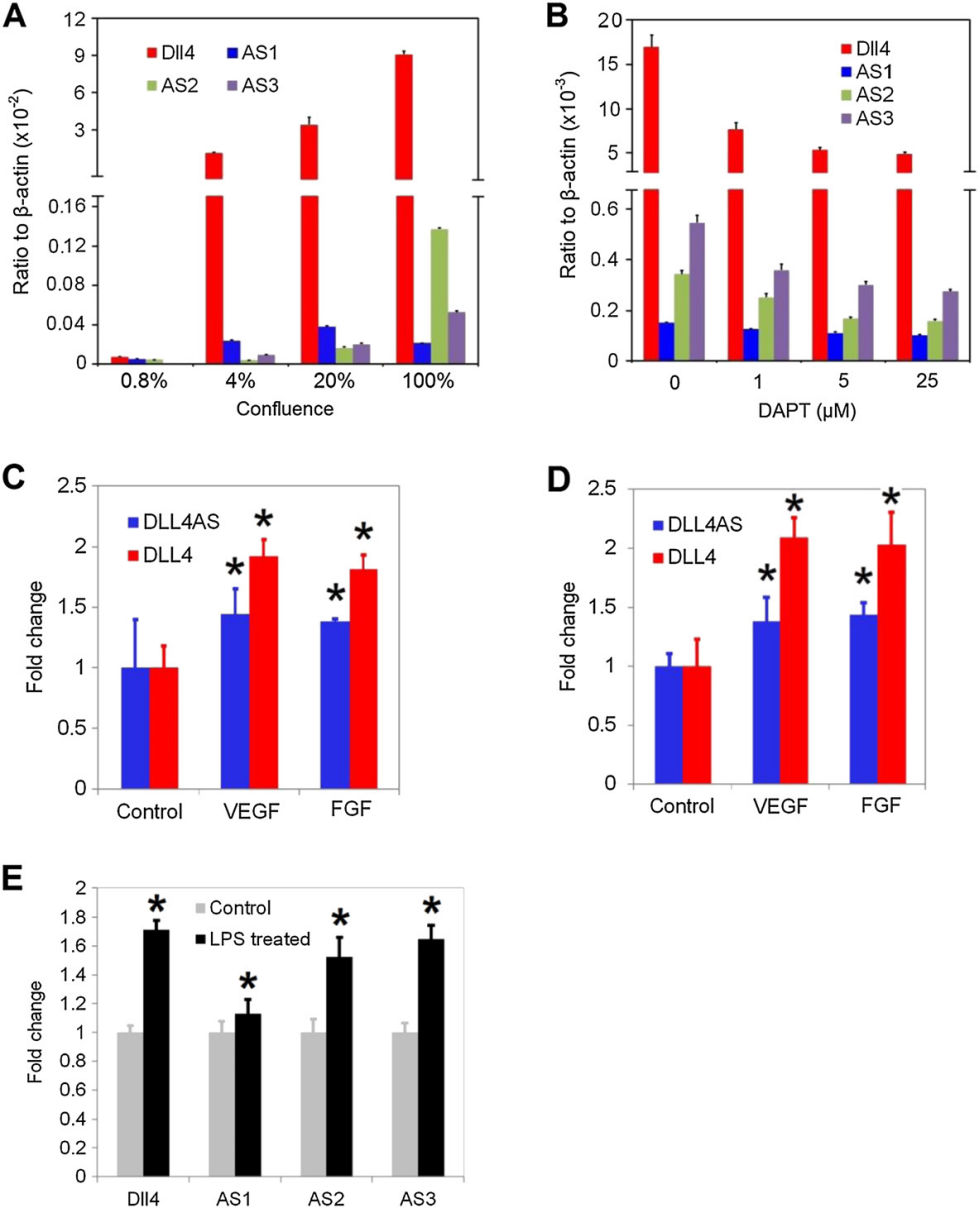
***Dll4-AS*** **responses to exogenous stimuli. (A)** *Dll4-AS* expression coincides with *Dll4* in the status of different cellular confluences. *Dll4-AS* expression level was measured in 0.8%, 4%, 20% and 100% confluent MS1 cells by quantitative PCRs. **(B)** DAPT treatment down-regulates the expression of both *Dll4-AS* and *Dll4* in MS1 cells. **(C)** HUVECs deprived of serum for 16 h were treated with 50 ng/ml recombinant human VEGF or FGF for 24 h. *DLL4* and *DLL4-AS* were quantitated by qPCR in these cells. **(D)** HCAECs deprived of serum for 16 h were treated with 50 ng/ml recombinant human VEGF or FGF for 24 h. *DLL4* and *DLL4-AS* were quantitated by qPCR in these cells. **(E)** 6-day old C57/BL6 mice were treated with 1 mg/kg intraperitoneal LPS. *Dll4* and *Dll4-AS* were quantitated by qPCR in neonatal mouse lungs 18 hr after systemic LPS. *denotes *p* < 0.05.

Because Notch-Delta signaling pathway has been extensively implicated in cell-cell contact signaling, we investigated the expression of *Dll4-AS* and *Dll4* mRNA levels in different confluent states of MS1 cells. Cells in 100% confluent state (dense) showed high levels of *Dll4-AS*, and *Dll4* mRNA levels, and this increase was observed as the confluency increased from 4-100%. Consistently, we observed three things. First, that *Dll4* expression is a log higher than *Dll4-AS*. Second, differences are observed within each of the three *Dll4-AS* transcripts across confluency stages, and third that both RNA levels change positively (Figure 3A). To investigate whether *Dll4* and *Dll4-AS* RNAs are co-regulated, we treated 90% confluent MS1 cells with increasing concentrations of DAPT, a notch inhibitor (Figure 3B), or human umbilical vein (Figure 3C) or coronary artery (Figure 3D) ECs with growth factors VEGF and FGF. In each case, we observed that when *DLL4* RNA level increases or decreases, the *DLL4-AS* levels increase or decrease respectively.

To confirm these findings in vivo, we performed experiments in the lung tissues in mice. Mice were treated with lipopolysaccharide (LPS) and lung tissues were harvested to analyze levels of *Dll4* and *Dll4-AS* RNA. LPS is known to induce angiogenesis and modify notch signaling [12,13]. Lung tissues isolated from LPS-treated neonatal mice showed increase in both *Dll4* and *Dll4-AS* RNA levels (−2 and −3) when compared to control mice (Figure 3E). The increase in *Dll4-AS* is less than that of *Dll4*, which is consistent with the data from cultured cells. These results suggest that *Dll4AS* regulation occurs both in vitro and in vivo, and the regulation is conserved across humans and mice presumably by a set of factors that bind at the common promoter region.

### *Dll4-AS* regulates *Dll4* expression

**Figure 4:**
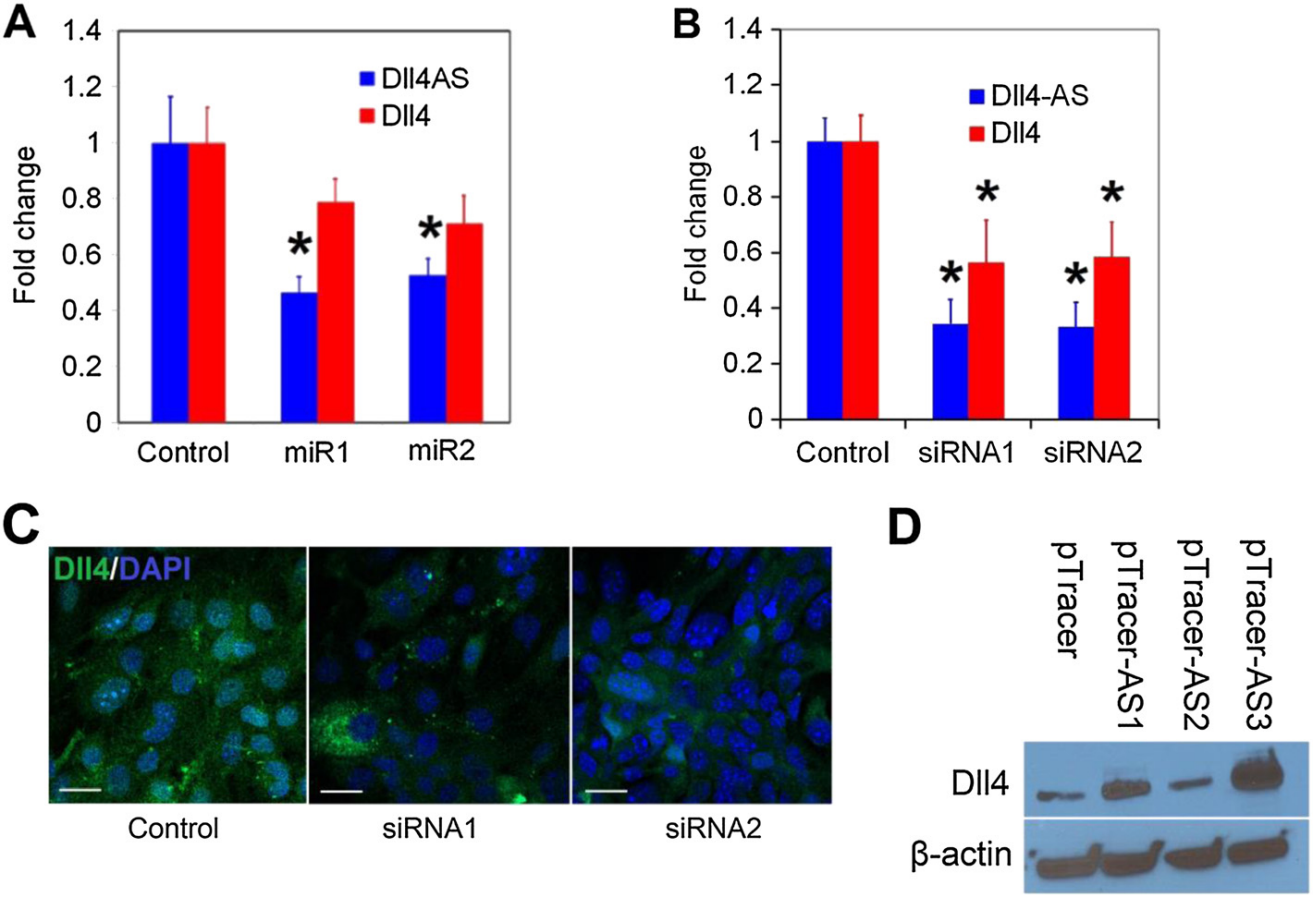
**Manipulation of** ***Dll4-AS*** **changes the expression of** ***Dll4*** **. (A)** pcDNA6.2-GW/EmGFP-*Dll4-AS* miR vectors were transfected into MS1 cells. qPCR was performed 48 h later. Synthetic siRNAs were transfected into MS1 cells. qPCR **(B)** and immunostaining **(C)** were performed 48 h later. pTracer-Dll4-AS vectors were transfected into MS1 cells. Western blotting **(D)** was performed 48 h later.

We investigated using loss-of-function approaches, the effect of *Dll4-AS* on *Dll4* RNA levels in ECs. We used pcDNA6.2-GW/EmGFP-miR vector designed to express artificial microRNAs (miRNAs). Two artificial miRNAs that showed 100% homology to the target sequence and targeting the common regions of *Dll4-AS* isoforms were designed. MS1 cells transfected with the two artificial miRNAs successfully decreased *Dll4* mRNA level (Figure 4A). To exclude the off-target possibility of the miRNAs, we used synthetic short hairpin silencing RNAs (siRNA) oligonucleotides targeting the common region of *Dll4-AS* isoforms. When the siRNAs were transfected into MS1 cells, *Dll4-AS* expression dropped 60%. Similarly, *Dll4* mRNA decreased roughly 40% (Figure 4B), which was further validated at the protein level by immunocytochemistry (Figure 4C). In gain-of-function experiments, we overexpressed individual *Dll4-AS* in MS1 cells. Western blot analysis of MS1 cell lysates showed that *Dll4-AS1* and -*AS3* up regulated the DLL4 protein levels (Figure 4D).

### *Dll4-AS* regulates angiogenesis

**Figure 5:**
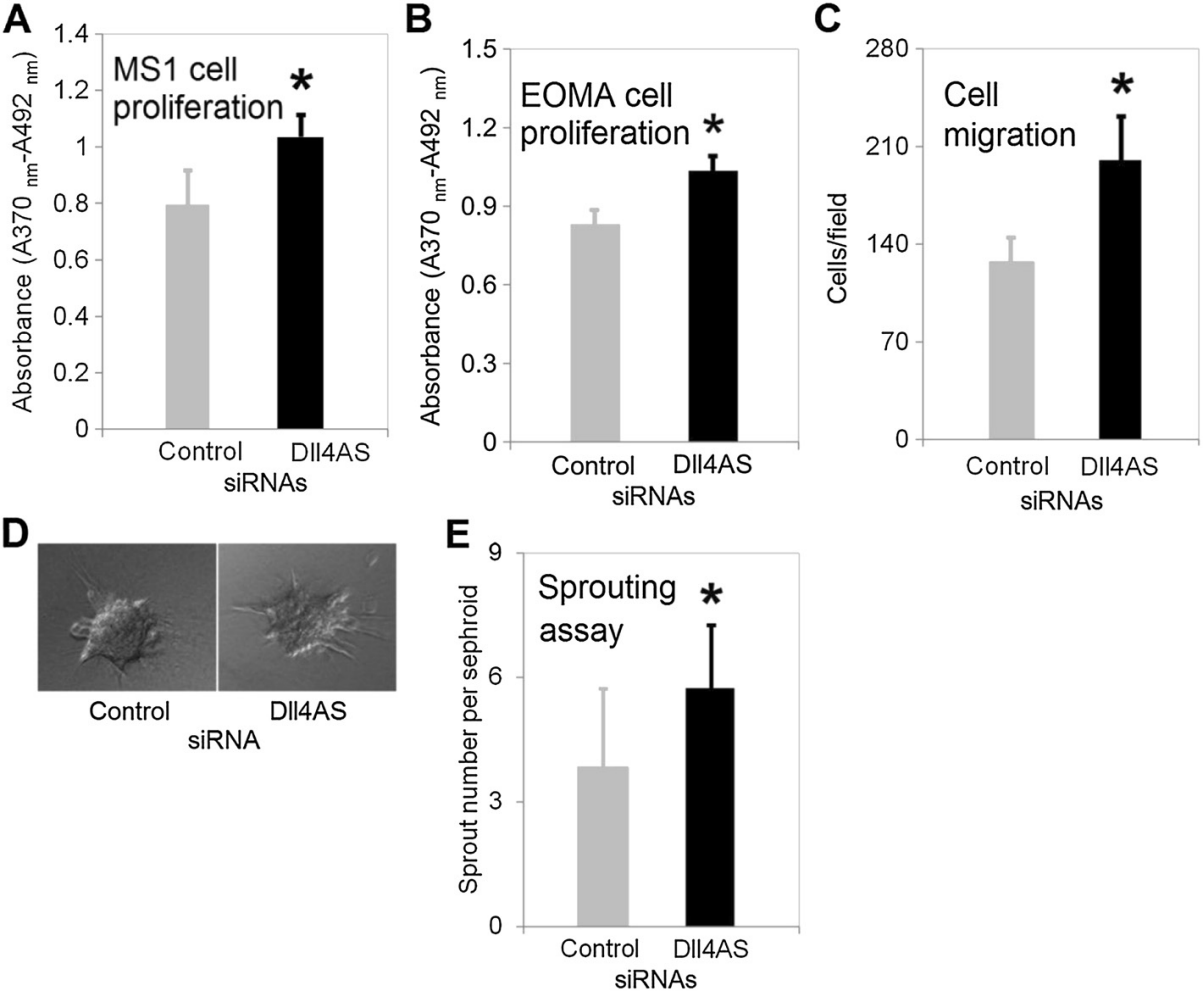
**Cell proliferation and migration assays. (A)** MS1 cells in 96-well plate were transfected with 1 pmol synthetic siRNAs. 48 h later, transfection medium was replaced with regular medium containing BrdU labeling reagent. Incubation lasted for 16 h before proliferation assay was performed. *denotes *p* < 0.05 **(B)** EOMA cells in 96-well plate were transfected with 1 pmol synthetic siRNAs. 48 h later, BrdU labeling solution was added into medium. Incubation lasted for 16 h before proliferation assay was performed. **(C)** Cell migration assay. EOMA cells in 6-well plate were transfected with 25 pmol synthetic siRNA. 48 h later, cells were placed onto trans-well membrane to allow migrate for 2 h. Cells were counted under microscope. **(D)** Representative spheroids with sprouting vessels. MS1 cells in 6-well plate were transfected with 25 pmol synthetic siRNA. 24 h later, cells were suspended in hanging drops to allow cellular aggregation. Spheroids were embedded in collagen gel to culture for 7 days. **(E)** Quantification of sprouts growing from the spheroids. 30 spheroids were examined for each transfection. *denotes *p* < 0.05.

Because *Dll4-AS* affects *Dll4* mRNA expression, we postulate that modulating *Dll4-AS* levels in ECs should affect EC phenotype. We focused on cell proliferation and migration, two phenotypes associated with tip vs. stalk cell formation where *Dll4* is known to participate in [14]. We compared the modulations of *Dll4-AS* in two cell lines namely MS1 and hemangioendothelioma (EOMA) cell line. EOMA cells are extensively used for vascular anomaly research, a condition associated with aberrant DLL4 signaling [15]. We performed BrdU-based proliferation assay in *Dll4-AS* silenced MS1 cells, and observed an increase in cellular proliferation (Figure 5A), which was also observed in EOMA cell line (Figure 5B). Intriguingly, *Dll4-AS* silenced EOMA cells showed increased migration to serum stimulus in the Boyden chamber assay (Figure 5C). To investigate *Dll4-AS* influence on sprout formation and branching, we generated cellular spheroids using MS1 cells. The spheroids were embedded in collagen gel and cultured under VEGF stimulation conditions. The sprouting vessels that emerged from the spheroids were analyzed at day 7. On average, 3.9 sprouts emerged from the spheroids of wild type MS1 cells, whereas 5.7 sprouts emerged from *Dll4-AS* knockdown [Figure 5D and E] MS1 cells. This data implies that similar to *Dll4*, *Dll4-AS* also restricts endothelial sprouting. These results collectively suggest that down-regulation of *Dll4-AS* and in turn *Dll4* impairs EC proliferation and migration responses, concepts associated with non-productive angiogenesis observed previously in *Dll4* knockout mice [16].

## Discussion

In this study, we have identified lncRNAs at the *Dll4* locus. The salient features of this study are: (a) Identification of three isoforms of *Dll4-AS* in murine ECs that are transcribed in the antisense direction to *Dll4*, and share the last exon; (b) Genomic locus of *Dll4* contains cis elements that are responsible for the dual transcription of *Dll4* and *Dll4-AS*; (c) Co-regulation of *Dll4* and *Dll4-AS* transcripts is observed in that loss of *Dll4-AS* affects *Dll4* mRNA level in vitro and in vivo; and (d) Down-regulation of *Dll4-AS* (and in turn *Dll4*) functionally impairs EC proliferation and migration, and enhances sprout formation. These results collectively implicate a new level of regulation at the *Dll4* gene locus in vascular development.

LncRNAs are a new class of regulators involved in genome organization and gene expression, especially in the process of cell differentiation and organ development [17]. In contrast to sncRNAs such as miRNAs, which target multiple coding sequences, lncRNAs usually target nearby genes [2,18]. Haploinsufficient genes like *Dll4* undergo tight regulation, and gene dosage is carefully monitored because *Dll4* is a key modulator of angiogenic sprouting and branching processes, critical events associated with physiological and pathological angiogenesis. Regulation of *Dll4* clearly occurs at the transcriptional and post-transcriptional levels [19]. Our report here proposes an additional layer of regulation to include lncRNAs-mediated *Dll4* regulation. Three lncRNA isoforms of *Dll4-AS* were identified with expression levels for each varying greatly across multiple cell types. The rationale for mutliple isoforms at this locus is unclear. Of the three isoforms of *Dll4-AS*, only *Dll4-AS1* overlaps with *Dll4*. We hypothesize that these RNAs are part of the checks and balances in the system for control of haploinsufficient gene expression.

Antisense ncRNAs arising from promoter regions can be classified into two categories according to their location [20]. The first category is composed of antisense ncRNAs overlapping with the corresponding mRNAs like *Dll4-AS1*. These antisense ncRNAs have been shown to down-regulate the corresponding mRNAs via the formation of ncRNA-mRNA duplexes [21]. The second category is antisense ncRNAs starting from regions upstream of the transcription start sites (TSSs) of the corresponding mRNAs, i.e., *Dll4-AS2* and *-AS3*. These antisense ncRNAs have been shown to functionally up-regulate the corresponding mRNAs via epigenetic mechanisms [22]. However, location of lncRNAs do not strictly dictate up or down regulation of cognate transcript. In our case, in MS1 and EOMA cells, *Dll4-AS2* and *-AS3* expression are highest compared to *Dll4-AS1.* Whether this selective up regulation of the AS isoforms is of functional consequence is yet to be determined. This selective up regulation was also noticed in the LPS-treated lung samples where AS2 and AS3 levels were up along with *Dll4*. Intriguingly, in overexpression experiments, *Dll4-AS1* and *AS3* transfected MS1 cells showed increased DLL4 protein compared to *Dll4-AS2*. Our results collectively suggest that selective combinatorial expression of *Dll4-AS* specific isoforms in cells and tissues control the expression of *Dll4*.

Genome locus of *Dll4* contains a number of cis elements, which allow transcription factors to bind and control the expression of *Dll4* [23]. *Dll4-AS* shares a common promoter region with *Dll4*, implicating a co-regulatory mechanism for both RNAs. Gene placement in a “head-to-head” fashion like that of *Dll4* and *Dll4-AS* is an ancient and conservative gene organization structure. The intergenic region between *Dll4* and *Dll4-AS* serves as a shared promoter, which drives the expression of the two genes toward opposite directions. This RNA Pol II-mediated process occurs in almost equal proportion in both directions [24]. The promoter sequence ultimately decides the dominant transcriptional direction [25], resulting in more abundant sense transcripts than antisense transcripts [24]. This is consistent in regards to the expression levels of *Dll4-AS* and *Dll4*, with more abundant *Dll4* mRNA observed in qPCR in cells, and also in reporter assays where the sense promoter direction is more active than the antisense direction. Most bidirectional promoters act as inseparable functional units that coordinately regulate the transcription of both genes [26], which is also consistent with our findings. The in vivo experiments on LPS-treated lung samples also confirm the co-regulatory aspects of this regulation.

Transcriptional correlation prognosticates functional association [27], so the function of *Dll4-AS* is likely to be pertinent to that of *Dll4*. In fact, both *Dll4-AS* and *Dll4* are co-expressed in ECs and show a positive correlation. Correlations between bidirectional transcripts could be positive or negative depending on differences in the cellular status [28]. Generally, positively correlated transcripts function in the same signaling pathway, and are coregulated in a common window of the cell cycle to respond to inductive signals [29-31]. Therefore, it is not surprising that down regulation of *Dll4-AS* downregulates *Dll4*. Further, down regulation of *Dll4* in mouse causes a hypersprouting phenotype [9]. However, these sprouts are non-functional, which is referred to as non-productive angiogenesis [16]. Similarly, when *Dll4* is downregulated due to loss of *Dll4-AS*, the ECs are hyperproliferative and hypermigratory, concepts that support non-productive angiogenesis. Sprouting was enhanced from *Dll4-AS* siRNA treated MS1 cells further confirming the similar functional role for *Dll4-AS* and *Dll4* in angiogenesis. Whether this regulation leads to differences in tip vs. stalk cell specification is unknown because Dll4-Notch is known to actively participate in this process [32]. Similarly, *Dll4-AS* and *Dll4* levels are affected by VEGF stimulation implying that *Dll4-AS* regulation of *Dll4* may participate in the VEGF-Notch cross talk pathway during angiogenesis. The factors that govern the regulation of the *Dll4-AS* transcript expression, and the role of antisense RNA regulation in specific processes of angiogenesis are all active areas of investigation in the lab.

## Conclusion

In summary, we report here the identification of three lncRNAs in the antisense direction in the *Dll4* locus. These antisense RNAs are co-regulated with *Dll4* RNA using a common genomic element. Downregulation of *Dll4-AS* affects *Dll4* levels in ECs, which in turn causes functional changes to the phenotype of ECs. We conclude that *Dll4-AS* regulation of *Dll4* is a novel mechanism of gene expression modulation, which has functional implications in vascular development.

## Additional file

Additional file 1: Figure S1. Schematic representation of *DLL4* locus in Homo sapiens chromosome 15, GRCh38 Primary Assembly. *DLL4* mRNA starting from 40929333 is shown in red boxes; *DLL4-AS* starting from 40927667 and ending at 40926900 is shown in a blue box.

## Abbreviations

Dll4: Delta-like4
Dll4-AS: Delta-like4 Anti-Sense
ncRNAs: Non-coding RNAs
lncRNAs: Long non-coding RNAs
VEGF: Vascular endothelial growth factor
FGF: Fibroblast growth factor
