# Supplementary material for: Delta-like 4 mRNA is regulated by adjacent natural antisense transcripts
Source: Vasc Cell. 2015 Mar 24;7:3. doi: 10.1186/s13221-015-0028-9 (PMC4409748; doi:10.1186/s13221-015-0028-9)
Supplement: Additional file 1: Figure S1. — Schematic representation of DLL4 locus in Homo sapiens chromosome 15, GRCh38 Primary Assembly. DLL4 mRNA starting from 40929333 is shown in red boxes; DLL4-AS starting from 40927667 and ending at 40926900 is shown in a blue box. [file 13221_2015_28_MOESM1_ESM.pdf]

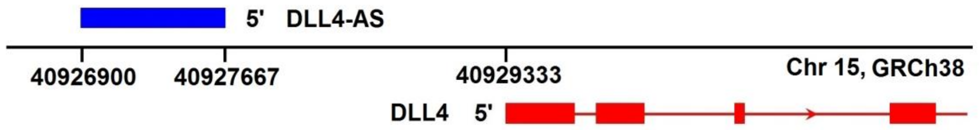

Supplementary figure 1 Schematic representation of *DLL4* locus in Homo sapiens chromosome 15, GRCh38 Primary Assembly. *DLL4* mRNA starting from 40929333 is shown in red boxes; *DLL4-AS* starting from 40927667 and ending at 40926900 is shown in a blue box.

DLL4-AS sequence

GGCCCAGGCTGCGCTCGGGAGTTAATGTAGGTTATGGGCAGGCGGAGTCCCAGGCCCGA
GGGAGGGGGAGTCCGGGGCGTGCCTCCCGGCGATCCTGGCAAGTGGGCTCCCAGATCTT
CTCTCCGCCTATACCCTATGGATCCCTGGACTTGCCTCTCTAAGATGCCAGCGGGTGCCC
CTTGCTCCCCTCCAAGCTGGCATCCAGCCCTACCTAGCCCTGTCCTCTCTCAACTCCAAAT
CCTGGCCCAGGAGTTAATAAAAACATCCTCCTTTTCTGGCGCTGGTCCTGCTTACCTGACC
CTCAAGGGAGACATCTAGTCCCACACAAGGCATCTCTCCCAATACTGGAAAATCTGGAACA
CTCTCTACCCTCATACACGCCCAGACTAATGTGCCCAAATGTACTTTGCAAATTATCAAGGA
CCAAACAAAAGTTGGGATTATTATCTTCAGAGAGGCAGCACCCAGGAGATCCCAGAGATCT
AGAAAGGCTCTGACCAGAAGAGGAAAGGGTTAAATTCTCAGCATCAAAAGACATTCATGAA
ACACCTATTTGCGGAGGCGCTGTCCCAGAAGCTCATTCCCGGGCTAGGACACTGCCACCC
TTGGCCCTGGGTACCTACGAGCGCTCCCACACGCTGCGGTCGAGCTTTGCCCACATTTGC
TGAGCAGGAGAGGGTCATGCTGCGGGTGGGGAGCCCAGTGCGAAGGCCGCAAGTGTTTC
TGTCAGCCCAAAACTGGGGCTGCGCCTTCCTTCTTCTCCCACTC
